# Drought, temperature, and moisture availability: understanding the drivers of isotopic decoupling in native pine species of the Nepalese Himalaya

**DOI:** 10.1007/s00484-024-02647-z

**Published:** 2024-03-05

**Authors:** Sugam Aryal, Jussi Grießinger, Narayan Prasad Gaire, Tribikram Bhattarai, Achim Bräuning

**Affiliations:** 1 Institute für Geographie, Friedrich-Alexander‐Universität Erlangen‐Nürnberg, Wetterkreuz 15, 91058 Erlangen, Germany; 2 Department of Environment and Biodiversity, University Salzburg, Salzburg, Austria; 3 Department of Environmental Science, Patan Multiple Campus, Lalitpur, Nepal; 4 Central Department of Biotechnology, Tribhuvan University, Kathmandu, Nepal

**Keywords:** Stable oxygen isotopes, Native pine species, Nepalese Himalaya, Precipitation, Temperature, Isotopic decoupling, Local environmental effects

## Abstract

The Himalayas experienced long-term climate changes and recent extreme weather events that affected plant growth and the physiology of tree species at high-elevation sites. This study presents the first statistically robust δ^18^O_TR_ chronologies for two native pine species, *Pinus roxburghii*, and *Pinus wallichiana*, in the lower Nepalese Himalaya. The isotope chronologies exhibited 0.88‰ differences in overall mean isotope values attributed to varying elevations (460–2000 m asl). Comparative analysis of climate response using data sets from different sources and resolutions revealed the superiority of the APHRODITE (Asian Precipitation – Highly-Resolved Observational Data Integration Towards Evaluation) data set calibrated for the South Asian Summer Monsoon (SASM)-dominated region. Both species exhibited negative correlations with monsoon precipitation and positive correlations with temperature. However, during the peak monsoon season (July-August), daily resolved climate data disentangled statistically insignificant relationships, and revealed that δ^18^O_TR_ is influenced by atmospheric moisture. Both congeneric species showed a decoupling between the chronologies after 1995. However, no significant change in air moisture origin and monsoon regime between the study sites was observed, indicating a consistent dominant moisture source during different monsoon seasons. Besides, we also observed the decreased inter-series correlation of both δ^18^O_TR_ chronologies after 1995, with *P. wallichiana* experiencing a steeper decrease than *P. roxburghii*. The weakening correlations between and within the chronologies coincided with a regional drought during 1993–1995 in both sites, highlighting the strong regulation of local climate on the impact of regional extreme climate events. Our findings emphasise the importance of employing climate data with optimal spatial and temporal resolution for improved δ^18^O_TR_-climate relationships at the intra-annual scale while considering the influence of site-specific local environmental conditions. Assessing climate data sets with station data is vital for accurately interpreting climate change’s impact on forest response and long-term climate reconstructions.

**Supplementary Information:**

The online version contains supplementary material available at 10.1007/s00484-024-02647-z.

## Introduction

The central Himalayan region, including Nepal, is experiencing a rapid positive temperature trend exceeding the global mean temperature increase (Shrestha and Aryal 2011). This warming can be associated with a shift in hydro-climate and changes in the frequency and severity of extreme precipitation events (Talchabhadel et al. 2018). Extreme climatic events are known to play a significant role in current and future ecosystem dynamics (Gottfried et al. 2012; Rangwala and Miller 2012). Climate change has already been observed as a key factor driving changes in species distribution, population structures, ecological zone shifts, species composition of ecosystems, phenology, and growing season on a local, regional, and global scale (Carrer et al. 2016; Gaire et al. 2017; Shrestha et al. 2012; Singh et al. 2016; Xu et al. 2009). Knowing the relationship between long-term environmental change and plant physiological responses is essential to better understand their current status and predict future scenarios. Studying tree-ring oxygen isotope variations on inter and intra-annual temporal resolutions can help to deepen insights into plant physiological and ecological responses to changing climate at different scales (Gessler et al. 2014; Szejner et al. 2018, 2020).

The oxygen isotope composition of tree-ring cellulose (δ^18^O_TR_) mainly depends on the isotopic composition of the source water, influenced by atmospheric moisture (McCarroll and Loader 2004). The isotopic composition of precipitation (δ^18^O_precip_) determines the δ^18^O of the source water at a given site. The δ^18^O_precip_ is further modified by temperature (temperature effect) and precipitation amount (amount effect) (Risi et al. 2008; Weatherford and Cummings 2016). The δ^18^O_TR_ additionally depends on fractionation effects at the leaf level. There, transpiration through the stomata leads to an enhanced loss of lighter water isotope molecules (^16^O), thereby modifying the δ^18^O composition of leaf water, followed by the modification of source water (xylem water) (Farquhar et al. 2007). Stomatal conductance is mainly controlled by relative humidity and water vapour pressure deficit (Dongmann et al. 1974; Farquhar et al. 2007; Szejner et al. 2016), resulting in higher enrichment of ^18^O during phases with high vapour pressure deficit (Kahmen et al. 2011; Sheshshayee et al. 2005). At the leaf level, additional fractionation occurs due to the advection of non-enriched xylem water to the evaporative sites opposing the back diffusion of the δ^18^O enriched water to the leaf lamina (Péclet-effect) (Barbour et al. 2004). However, the δ^18^O modification due to the Péclet-effect is usually negligible compared to the evaporative enrichment (Song et al. 2014). According to previous experiments, on average, an enrichment factor of 27‰ is considered for cellulose δ^18^O relative to the source water (Da Sternberg et al. 1986; Luo and Da Sternberg 1992). Hence, sucrose formed during assimilation (exchange of oxygen atoms between enriched leaf water and carbonyl groups) records the ^18^O signature of the leaf water. The sucrose is carried down along the trunk through the phloem to the cambium cells. During cellulose synthesis, oxygen atom exchange occurs between sucrose and source (xylem) water (Sternberg et al. 2006), which may lead to an exchange of 43% of all oxygen atoms (McCarroll and Loader 2004). Thus, the isotopic signal of cellulose dominantly reflects the δ^18^O of the source water and leaf water enrichment.

Several studies focused on the analysis of tree-ring δ^18^O_TR_ from high-elevation conifers to understand their adaptation to possible long-term and seasonal environmental changes in the Himalayan region (Bose et al. 2016; Brunello et al. 2019; Managave et al. 2020; Sano et al. 2010, 2011, 2013; Xu et al. 2018) and from the adjacent areas of Tibetan Plateau and Southwest China (Yunnan province) (Fan et al. 2020; Fu et al. 2016; Grießinger et al. 2019; Hochreuther et al. 2016; Huang et al. 2019; Wernicke et al. 2017; Xu et al. 2016). However, studies focusing on lower-elevation conifer species are still underrepresented, although the middle mountain forest belt forms an important resource for the regional ecosystems and peoples’ livelihoods. Previous studies have reported a contrasting δ^18^O_TR_-climate response along elevation (Brunello et al. 2019) and latitudinal (Managave et al. 2020) gradients due to the complex topography of the Himalayas, demanding further regional studies to increase the density of existing isotope networks. The impact of changing precipitation seasonality, seasonal water availability, and enhanced exposure to droughts on plants is primarily attributed to varying monsoon dynamics. However, little information is available about the long-term monsoon variability in the Himalayas due to the lack of a dense network of instrumental climate stations (Sano et al. 2020).

Notably, δ^18^O_TR_ series on a regional scale can reveal a strong coherency within and between species (Baker et al. 2015; Grießinger et al. 2019; Loader et al. 2019). On the other hand, contrasting relationships between isotope series and climate were also reported worldwide. Recently, a study comparing δ^18^O_TR_ series across the globe pointed to a detectable inconsistency in the δ^18^O_TR_-climate relationship (a so called “isotopic-divergence” phenomenon) (Savard and Daux 2020). Climate change was imputed as being the most important driving factor coupled to a shift in moisture origin, an increase in drought events accompanied by lowered annual soil water pools, pollution effects, and/or a shift in monsoon timing/season (Savard and Daux 2020). In addition, even δ^18^O_TR_ chronologies of two or more species can show different or contrasting responses to extreme climate events based on their physiological adaptation strategy or varying micro-site conditions (Altieri et al. 2015). Such species-specific adaptation strategies can sometimes result in contrasting δ^18^O_TR_ trends (hereafter termed as “isotopic decoupling”).

Within this study, we analysed the possible effects of a changing climate on the δ^18^O_TR_ variations in two native pine species from the foothills of the Nepalese Himalayas. *Pinus wallichiana* (A. B. Jackson) is adapted to a temperate climate, ranging from 1600 m a.s.l. up to the tree line (> 3800 m a.s.l.) (Eckenwalder 2009). On the other hand, *Pinus roxburghii* (C. S. Sargent) is a subtropical conifer species distributed in the middle mountain belt in the Himalayas from 400 to 2300 m a.s.l. (Farjon 2011). Both pine species generally form pure stands on southerly exposed slopes. According to the forest cover map of Nepal, both pine species combined represent 10.80% of the available total stem tree volume (Department of Forest Research and Survey 2015). They notably provide high-quality timber and non-timber forest products (NTFP) for the local socioeconomy, for example resin that is collected for purposes of the chemical industry (Chauhan et al. 2022). The NTFPs, like pine resin, are of crucial benefit for livelihood by uplifting the local to regional economic status (Paudyal 2010). However, the resin production depends on the trees’ vitality and their physiological adaptation to extreme climate events associated with ongoing climate change. Therefore, it is crucial to understand how trees perform under rapidly changing environmental conditions to cope with climate change and extreme events such as drought. Recent studies on tree-ring width reported the sensitivity of both species for recording pre-monsoon precipitation(Aryal et al. 2018; Gaire et al. 2019; Misra et al. 2021; Sigdel et al. 2018). This study presents a novel analysis of trees from the middle elevation belt, comparing δ^18^O_TR_ of two pine species in the monsoonal-influenced central parts of the Himalayas. We envisaged this research to discover and compare the plant’s physiological performance with changing climate using tree-ring δ^18^O_TR_ along an elevation gradient. To better compare δ^18^O_TR_ chronologies and their response to seasonal/annual environmental changes, we hypothesised that being congeneric and growing under the strong influence of the South Asian Summer Monsoon (SASM) climate, isotope chronologies of both species show a similar moisture signal. However, elevation and local moisture conditions control the strength and possible change of the regional climate effect. Our hypothesis is based on the following research questions: (a) which climate parameters during which season influence the δ^18^O_TR_ of both studied pine species, (b) is the response of δ^18^O_TR_ to climate and their coherency between species consistent over time, and (c) If not, what causes the observed decoupling.

## Materials and methods

### Study area and climate

**Fig. 1:**
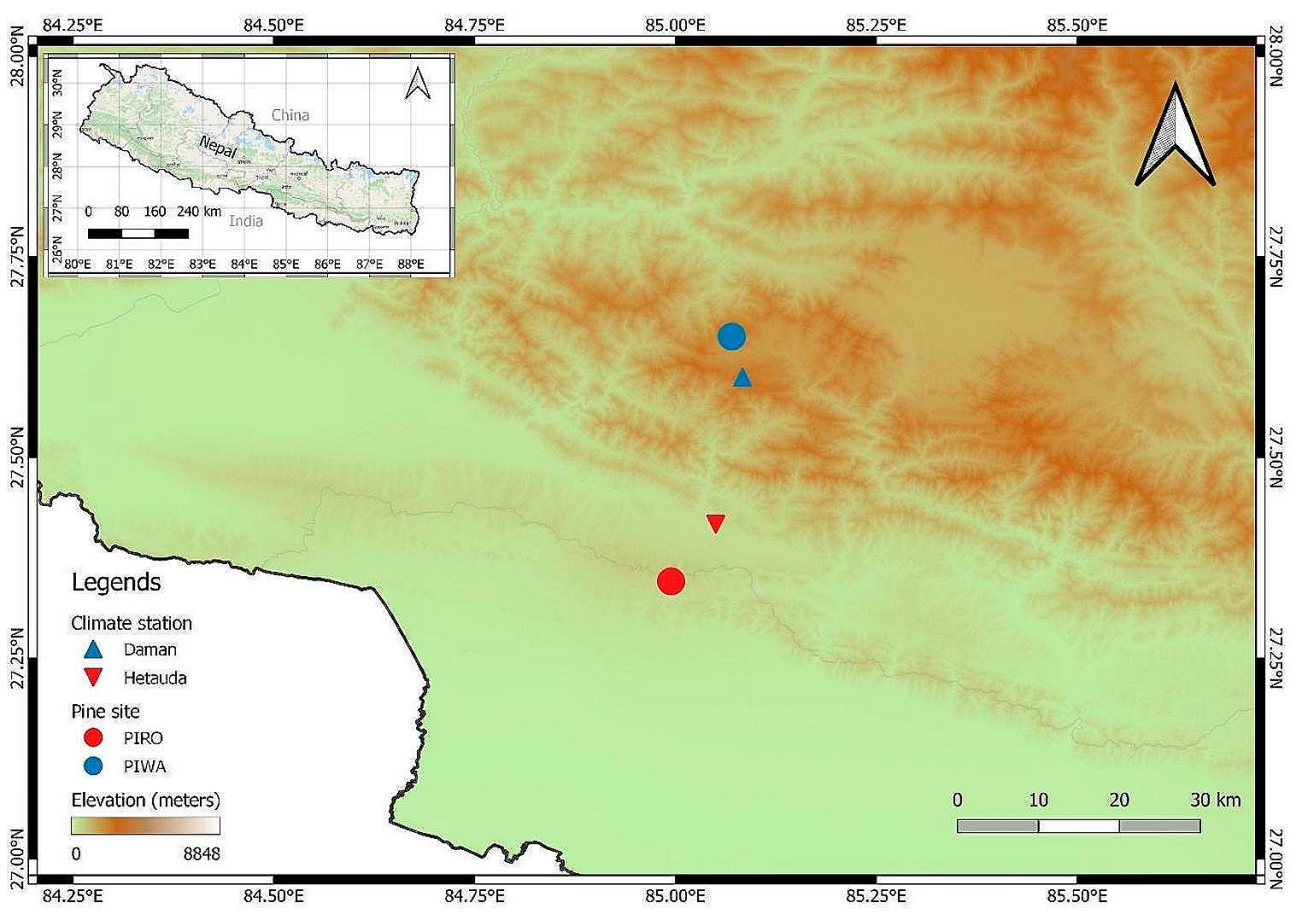
Map of the study area indicating study sites (blue and red circles), climate stations (blue and red triangles), and elevation (colour in the background)

Our study was conducted in two different altitudinal settings (Fig. 1) in central Nepal. The lower elevation site, PIRO (27.37^0^ N, 85.03^0^ E), was located in the Chure range at about 460 m a.s.l. The upper elevation site, PIWA (27.65^0^ N, 85.06^0^ E), was situated in the Mahabharat range at about 2000 m a.s.l. Both study sites illustrate the lower species distribution limits for the respective species.

**Fig. 2:**
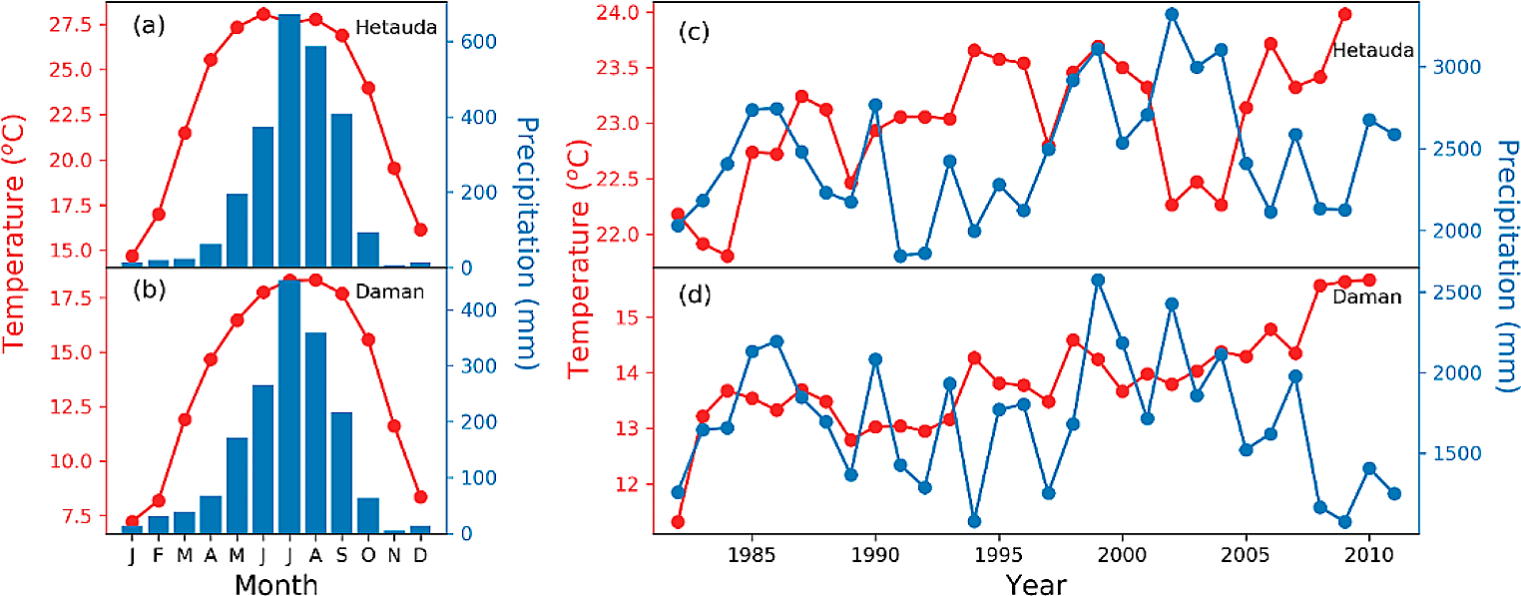
Average climate conditions and recent climate trends in both study sites. (**a**) and (**b**) represent the monthly temperature and precipitation patterns in the Hetauda and Daman stations. (**c**) and (**d**) describe the annual mean temperature and precipitation trends —source: Government of Nepal, Department of Hydrology and Meteorology

Our study sites are influenced by the Indian summer monsoon, with maximum temperature and precipitation occurring during the summer monsoon season (June-September). A tropical savannah climate characterises the PIRO, whereas the PIWA site has a temperate mountain climate with dry winters and hot summers (Karki et al. 2016). The nearest climate stations to PIWA and PIRO sites were Hetauda and Daman, respectively. The 30-year-long observed climate data show an unimodal monthly climate trend, with June-August being the warmest and wettest season of the year (Fig. 2). The maximum average temperature in Hetauda is 10^O^ C warmer compared to the Daman station. Likewise, the mean total monthly precipitation for Hetauda is almost 200 mm higher compared to Daman. A trend analysis of the mean annual temperature from 1982 to 2012 reveals an overall increase in mean temperatures for both sites. However, the rate of warming is higher in the higher elevation (PIWA) site, confirming elevation-dependent climate change (Pepin et al. 2015). Precipitation does not show any long-term trend in both sites. However, there was a steep decrease in rainfall in Daman after 2000 CE (Fig. 2). The steep decrease in precipitation after 2000 CE, coupled with increasing temperature, points towards the aggravation of the water balance due to high evapotranspiration (also shown by SPEI4 in Fig. S9).

### Sample collection and processing

For this study, we selected two pine species growing at two different elevations, i.e., *P. roxburghii* at site PIRO and *P. wallichiana* at site PIWA (Table 1). We collected two cores from 10 trees at breast height (1.3 m) using an increment borer five mm diameter. The samples were air-dried and then polished using sanding papers of different grit sizes to make tree-ring boundaries clearly visible. Subsequently, tree-ring widths (TRW) were measured using a LINTAB system operated by TSAPwin software (Rinn 2013). First, the TRW series of two radii of the same tree were cross-dated. Then, the mean series of different trees were cross-dated with each other. We considered Gleichlaeufigkeit (Glk), Crossdate Index (CDI) (Rinn 2013), and inter-series correlation as basic statistics for cross-dating to evaluate the reliability of our TRW chronologies.

**Table 1:** Site description and time series statistics of the δ^18^O_TR_ and TRW chronologies. Abbreviations: $\stackrel{-}{\varvec{r}}$ = mean inter-series correlation; SD = standard deviation and AC1 = first-order autocorrelation. The means and standard deviations of raw TRW chronologies are presented in the table

Site	Species	Elevation (m a.s.l.)	Number of core		Span		$\stackrel{-}{\varvec{r}}$		Mean ± SD		AC1	
			δ^18^O_TR_	TRW	δ^18^O_TR_	TRW	δ^18^O_TR_	TRW	δ^18^O_TR_ (‰)	TRW (mm)	δ18O_TR_	TRW
PIRO	*P. roxburghii*	460	6	14	1947–2014	1946–2014	0.74	0.61	28.07 ± 1.06	3.31 ± 1.90	0.11	0.42
PIWA	*P. wallichiana*	2000	7	19	1944–2014	1944–2014	0.70	0.46	27.08 ± 1.37	4.19 ± 2.51	0.08	0.47

Out of the overall sample pool of 20 increment cores per site, we chose the seven best correlating samples from each species for the subsequent stable isotope analysis. In a second step, each annual tree ring was separated under a binocular using a razor blade and stored in an Eppendorf tube. Alpha-cellulose of each sample was extracted following the method suggested by Wieloch et al. (2011). We then homogenised the alpha-cellulose using an ultrasonic device (Laumer et al. 2009. The homogenised samples were freeze-dried, weighed, and packed in a silver capsule. Isotope values were measured in a ratio mass spectrometer (Delta V Advantage, Thermo Fisher) coupled to a high temperature (1450 °C) pyrolysis reactor (HT Oxygen Analyzer) with an analytical precision of ± 0.25‰.

The TRW and stable isotope chronologies were further investigated by calculating the overall and running inter-series correlations using the ‘dplR’ package. We used six and seven series for isotope chronologies of *P. roxburghii* and *P. wallichiana*, whereas for TRW chronology, 14 and 19 series of PIRO and PIWA were used, respectively.

### Climate data and statistical analysis

The instrumental climate data recorded from nearby stations were short (only available from 1982 to 2012) for performing robust statistical analyses. Therefore, gridded or reanalysis climate data products from various sources were used. In addition, the complex mountainous topography is characterised by strong spatio-temporal heterogeneity affecting temperature and precipitation (Karki et al. 2016). It is crucial to validate gridded or reanalysis climate data against the observed climate to get suitable data that better capture spatial and temporal patterns and represent microclimate conditions (Chen et al. 2021; Kanda et al. 2020) that imprint extreme climate events like drought. For this, we used climate data with different spatio-temporal resolution for further statistical analyses like CRU TS 4.04 (1961–2014 CE) (Harris et al. 2020), ERA5-land (1981–2014 CE) (Copernicus Climate Change Service 2019), and APHRODITE (1961–2014 CE) (Yatagai et al. 2012). The spatial resolution of CRUTS, APHRODITE, and ERA5 data sets are 0.5^°^, 0.25^°^, and 0.1^°^, respectively each with monthly, daily, and hourly resolutions. The robustness of the chosen data set was tested with instrumental data from nearby stations of each study site using simple Pearson correlations. We used daily precipitation and temperature data from the APHRODITE data set to analyse the effect of short-term environmental change on our annual δ^18^O_TRC_ data. The 65 (55 for temperature) years-APHRODITE data set provides a more robust correlation analysis than the available 32-year ERA5-land data set. Since the APHRODITE data set only provides average temperature and precipitation, we used the daily temperature and dew point temperature of the ERA5 data set to calculate daily vapour pressure deficit (VPD) and relative humidity (RH). We estimated the monthly VPD and RH using monthly temperature and vapour pressure following the method introduced by Huang et al. (2018) for CRU TS data. In total, we were able to extract nine climate parameters from the three mentioned data sets (Table 2). In our manuscript, we use the following acronyms to indicate the monthly climate variables for a better readability (cf., Table 2).

**Table 2:** The climate data set, their variables, and acronyms used to denote them

Data set	Temporal resolution	Spatial resolution	Variables	Acronyms
CRU TS 4.04	Monthly	0.5^°^	Temperature	cruTEM
			Precipitation	cruPRE
			scPDSI	cruscPDSI
			Relative humidity	cruRH
			Vapour pressure deficit	cruVPD
ERA5-land	Daily	0.1^°^	Temperature	eraTEM
			Precipitation	eraPRE
			Relative humidity	eraRH
			Vapour pressure deficit	eraVPD
APHRODITE	Daily	0.25^°^	Temperature	aphTEM
			Precipitation	aphPRE

We calculated bootstrapped Pearson’s correlation coefficients (r) between δ^18^O_TR_ and monthly and daily climate parameters to assess the isotope-climate relationship using the Scipy module (Virtanen et al. 2020) of the Python 3 programming language (van Rossum and Drake 2009). For the daily climate data, we first calculated 30 days moving averages (for temperature and VPD) or the sum (for precipitation). Then, we applied 30-year moving windows to calculate the correlation values in both sites and examined their consistency with changing temperature and precipitation trends. For the first 29 days, the 30-days moving average included days from previous years to current year. For example, the correlation coefficient on the day of the year (DOY) 1 represents the correlation of the mean or sum of daily climate from the DOY 336 of last year to the DOY 1 of the current year. All the analysis and graphical representation were performed using the Python 3 programming language (van Rossum and Drake 2009). We used piecewise linear regression to determine the onset and cessation of monsoon in study sites. First, the cumulative precipitation was calculated using daily precipitation data from the APHRODITE data set (Fig. S3) for each year. Then, we applied the “pwlf” module (Jekel and Venter 2019) to linearly fit three line segments (wx, xy, and yz) for each year’s cumulative precipitation (Fig. S3)(Cook and Buckley 2009). The first breakpoint is set to be x1 (the smallest x value in the dataset), and the last breakpoint is set to be xn (the largest x value in the dataset). The optimisation problem is solved by finding the locations of the other breakpoints (from x2 to xn-1) that minimise the overall sum-of-square of the residuals. The ‘Differential Evolution (DE)’ strategy was used for optimisation. This is the default optimisation strategy used in the ‘fit’ function of the “pwlf” library. DE is a popular global optimisation algorithm suitable for problems with multiple local minima and provides robust results (Jekel and Venter 2019). The starting and endpoints of the second line segment (xy) were considered to be the onset and cessation of the monsoon for each year.

### Back trajectory analysis

Local precipitation trends show strongly declining trends in recent years, especially after 2000 CE (Fig. 2). We, therefore, studied this period in detail by tracking the moisture sources using back trajectory analysis. The back trajectories were calculated using the NCAR reanalysis data set (Compo et al. 2010) using the HYSPLIT (HYbrid Single-Particle Lagrangian Integrated Trajectory) modelling system for trajectories, dispersion and deposition (Draxler and Hess 1998). We used the module ‘PySPLIT’ (Warner 2018), based on the Python 3 programming language, for faster and more efficient calculation. The five-day-long trajectories were developed for five-year windows from 1981 to 2015. The calculation was performed for two periods for each year of each five-year window. The first period was at the beginning of the summer monsoon (i.e., from 105 to 181 days of the year), and the second period was at the end of the summer monsoon (i.e., from 243 to 304 days of the year). Only those trajectories that caused precipitation of more than zero mm at the site and had an integration error of less than five were selected. The trajectory frequency was six per day: at 06:00, 12:00, and 18:00, each at 500 and 1500 above ground level.

## Results

### Chronology comparison

**Fig. 3:**
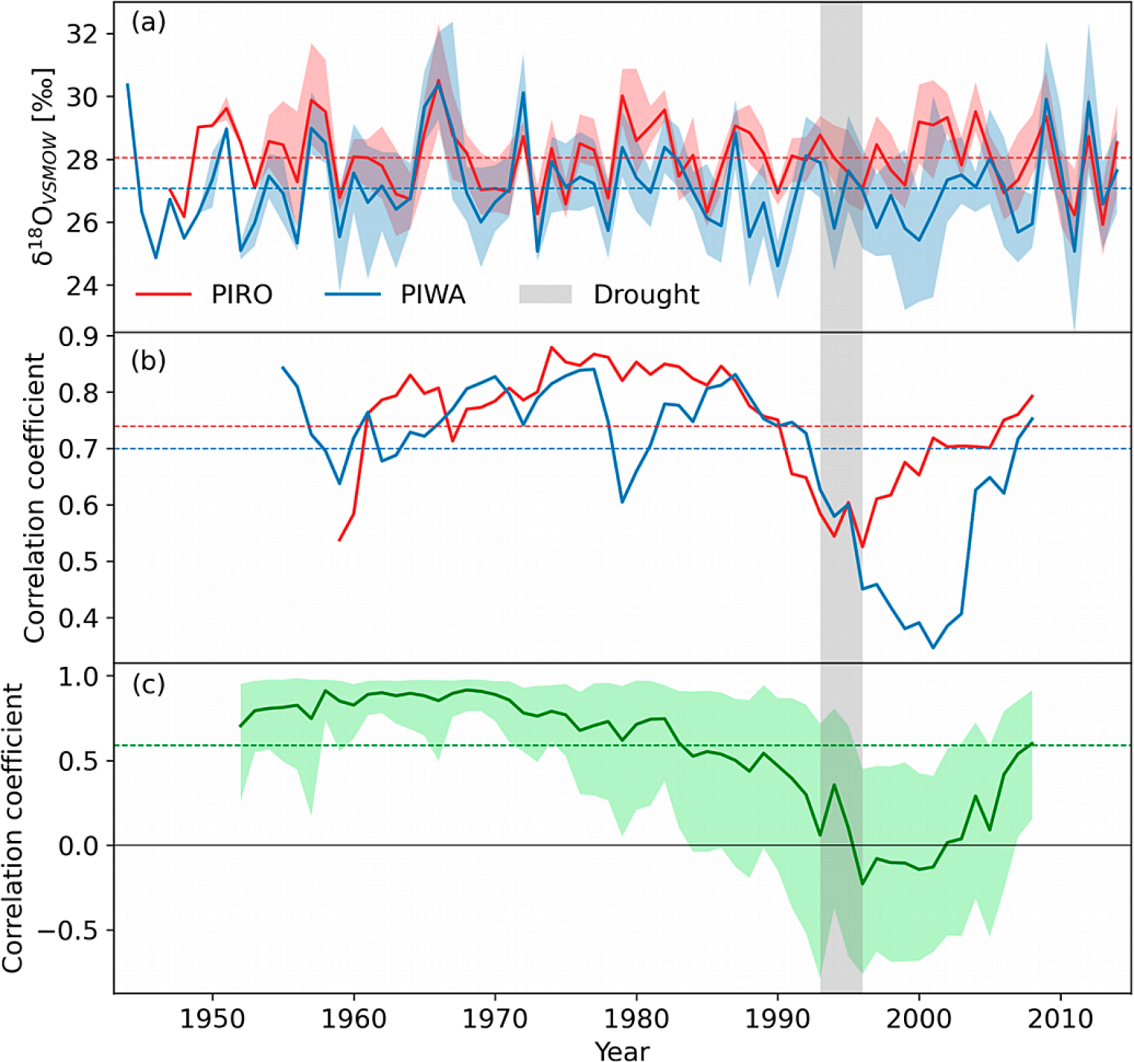
(**a**) Stable oxygen isotope chronologies of P. roxburghii and P. wallichiana, including the 5% confidence intervals and mean values represented by dashed coloured lines. (**b**) The 11-year moving mean inter-series correlation of the isotope series. (**c**) The 11-year running bootstrapped correlation between the PIRO and PIWA isotope series with a 5% confidence interval. The grey-shaded vertical column represents the drought period from 1993 to 1995

The inter-series correlation of both TRW chronologies increased during the 1990s, as did the correlations between the site chronologies (Fig. S1). The subsample signal strength (SSS) of both δ^18^O_TR_ was well above the threshold value (0.85) after 1954 CE, evidencing a robust common chronology signal (Buras et al. 2017). The average δ^18^O_TR_ of PIRO and PIWA were 28.07 ± 1.06‰ and 27.00 ± 1.37‰ (Table 1), and the mean inter-series correlation of both δ^18^O_TR_ chronologies was 0.74 and 0.70, respectively (Fig. 3). Both chronologies showed very low first-ordered autocorrelation, suggesting that they were free from any carry-over effects from the previous year (Table 1). The 11-year running inter-series correlation revealed a wiggly trend for both sites, with a decreased correlation from the mid-1980s to 2000 and a recovery in recent years (Fig. 3). However, the depression of the PIWA inter-series correlation was more pronounced in terms of both the correlation amount and the recovery duration. The two site δ^18^O_TR_ chronologies showed stronger coherency (*r* = 0.59, *p* < 0.001) with each other than TRW chronologies (*r* = 0.19, insifnificant). However, interestingly, the 11-year running correlation between the site chronologies followed the same trend as the running inter-series correlation, with an insignificant correlation after 1985 (Fig. 3b). The 11-year running correlation between TRW chronologies showed the opposite trend for the same period (Fig. S1). The inter-series correlation of the two TRW chronologies showed an increasing trend after 1985 (Fig. S1).

### Evaluation of isotope-climate relationships

**Fig. 4:**
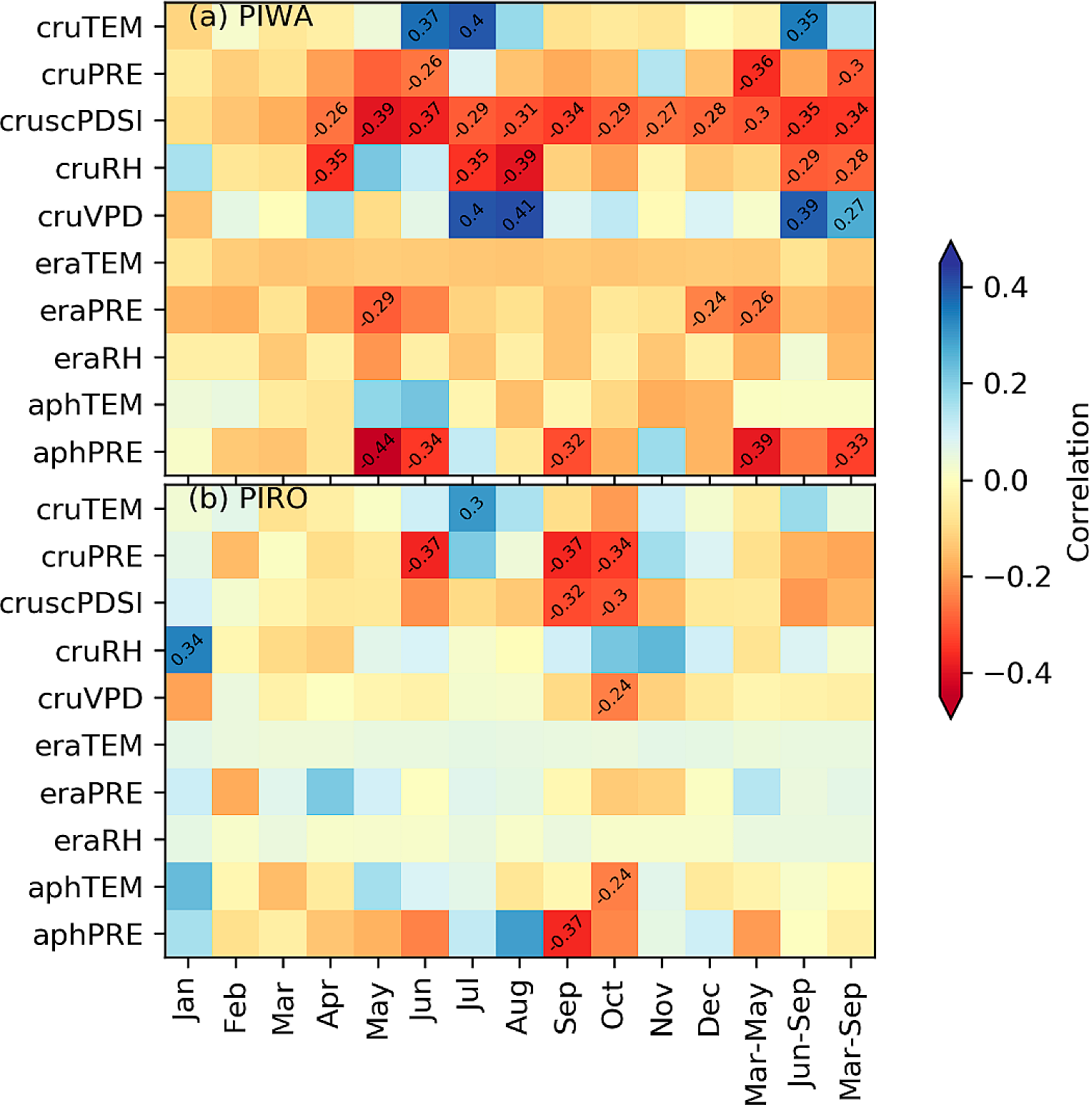
Correlation diagram between δ^18^O_TR_ chronologies of the two studied pine species and climate variables from CRU, ERA5, and APHRODITE data sets. TEM, PRE, scPDSI, and RH denote the average temperature, total precipitation, self-calibrated PDSI, and relative humidity. Cells including a value represent significant correlations at *p* < 0.05

Figure 4 displays the δ^18^O_TR_-climate relationship of both isotope chronologies with monthly temperature (TEM), precipitation (PRE), relative humidity (RH), and self-calibrated Palmer Drought Severity Index (scPDSI) derived from the CRU TS, ERA5-land and APHRODITE data sets. The climate variables directly related to moisture, such as precipitation, scPDSI, and relative humidity, showed a significant negative correlation with the PIWA chronology (Fig. 4a). The relationship with cruPRE and aphPRE was significant during the pre-monsoon (March-May) and growing seasons (March-September). In contrast, response with eraPRE was only significant for the pre-monsoon season. The cruscPDSI showed a strong relationship for May to December, which ultimately accounted for a significant correlation for pre-monsoon, monsoon, and the entire growing season. In relative humidity, cruRH showed a significantly negative response for monsoon and growing seasons. For temperature, the PIWA chronology showed a positive correlation in June and July with the cruTEM. However, the correlation with seasonal temperature was not statistically significant. In contrast, calculations with cruTEM, eraTEM, and aphTEM were insignificant.

Likewise, the PIRO chronology (Fig. 4b) also displayed a similar negative yet weaker relationship with cruPRE for the pre-monsoon and growing seasons. The correlation with aphPRE was only significant for September, without any strong relationship with seasonal precipitation. The eraPRE did not show any statistically significant association with PIRO chronology. With cruscPDSI data, the response was significantly negative for September, October. The cruRH for January correlated positively with PIRO chronology, but the effect of eraRH on the chronology was statistically neutral. The relationship with temperature was inconsistent with the different data sources. The July cruTEM correlated positively with the chronology. Unlike PIWA chronology, PIRO chronology was significantly linked with October aphTEM.

**Fig. 5:**
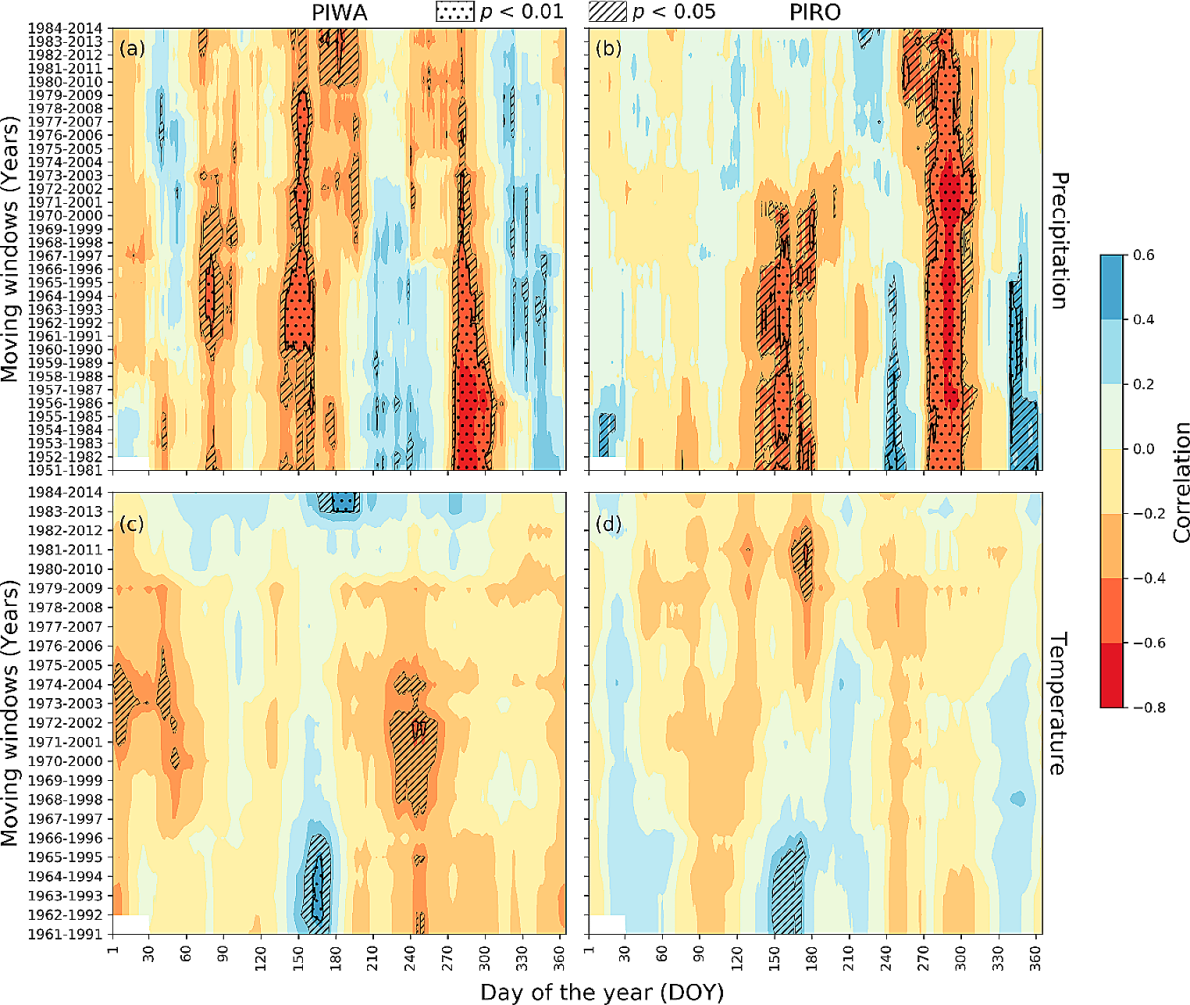
30-year moving correlation analysis of PIWA and PIRO chronologies using 30-day moving climate windows of APHRODITE data set. Figures (**a**) and (**b**) represent correlations with running sums of daily precipitation, (**c**) and (**d**) represent correlations with a running mean of daily temperatures. Shaded areas represent significant correlations at *p* < 0.01 (dotted areas) and *p* < 0.05 (hatched areas) levels, respectively

**Fig. 6:**
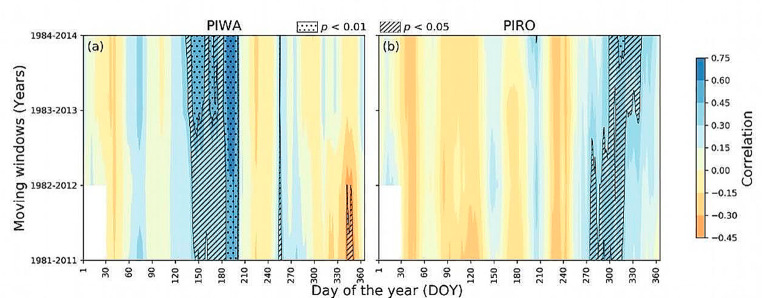
30-year moving correlation analysis of PIWA and PIRO stable oxygen isotope chronologies using 30 days moving averages of vapour pressure deficit (VPD) of (**a**) PIWA and (**b**) PIRO site using ERA5-land data set. Shaded areas represent significant correlations at *p* < 0.01 (dotted areas) and *p* < 0.05 (striped areas)

The relationships between daily APHRODITE climate and annual δ^18^O_TR_ are depicted in Fig. 5. Similar to the monthly climate data results, correlations with daily data show for the early study period (1951–1995 CE) consistently negative correlations of both δ^18^O_TR_ chronologies with early monsoon season precipitation (Fig. 5a and b). The correlation was more robust than with monthly climate, particularly from 105 to 181 days of the year. In contrast, the response to climate became insignificant after the first half of the 1990s at the PIRO site (Fig. 5b). On the other hand, the correlation with late-monsoon precipitation (DOY 243–304) remained consistently significant throughout the entire study period (Fig. 5b). However, the strength of the correlation was more stable in PIRO than in PIWA. Like precipitation, temperatures during the early monsoon season significantly correlated with the chronology before 1996 at both sites (Fig. 5c and d). This correlation disappeared in recent years at both elevations. The daily VPD showed stable positive relationships during the early-monsoon season (DOY 140–200) for PIWA and during the late-monsoon season (DOY 270–330) for PIRO (Fig. 6).

## Discussion

### Characteristics of isotope chronologies

The higher average δ^18^O value of the PIRO chronology than of the PIWA chronology can be attributed to differences caused by the “altitude effect”. The altitude effect is primarily governed by temperature. The elevation difference of 1500 m between the two sites implies cooler conditions in higher elevations, leading to a lower dew point temperature. As a result, the air moisture in higher elevations condenses faster than in lower elevations, preventing possible discrimination of rainwater δ^18^O (Gonfiantini et al. 2001). In a previous study, Brunello et al. (2019) found an average δ^18^O_TR_ of 26‰ in *P. wallichiana* from a high-elevation site in Nepal, about one parts-per-million (‰) depleted relative to our result. In the Himalayas, the rainwater δ^18^O lapse rate was reported to be 0.15‰ per 100 m, less than the global average lapse rate (0.28‰ per 100 m) (Wen et al. 2012). If interpolated on an elevation difference of 1500 m, this would mean a difference in the source water signal of 2.25‰, with lower values at the higher-elevation site. However, the mean δ^18^O_TRC_ value of our lower-elevation PIRO chronology is 0.88‰ higher than that of the PIWA chronology, implying stronger isotope discrimination at the low-elevation site, possibly by higher temperatures and VPD, leading to a stronger enrichment of ^18^O in the needles of PIRO.

### Effect of temporal and spatial resolution of climate data

**Fig. 7:**
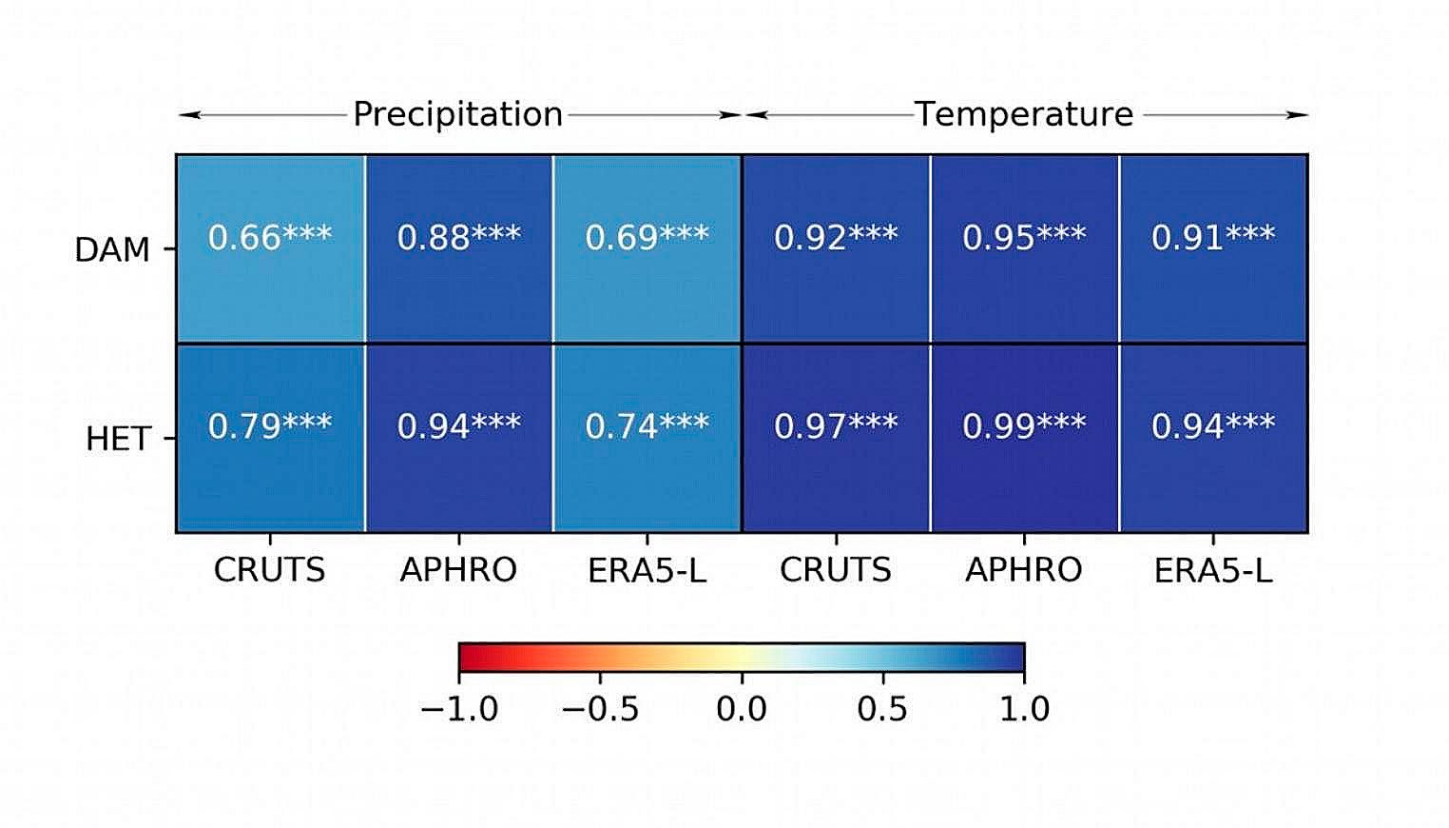
Comparison of linear regression coefficients between CRU TS, APHRODITE, and ERA5-land reanalysis data sets and instrumental climate data (1982–2012). DAM and HET represent the climate of the local meteorological stations at Daman and Hetauda. ‘***’ means indicates regressions significant at the 99% confidence level

Regarding monthly resolved climate data, we only used the climate variables commonly available in all data sets, i.e., mean temperature and precipitation for further discussion. Of all data sets used, the APHRODITE-based results for precipitation revealed the most significant correlations with δ^18^O_TRC_ chronologies (Fig. 4). In contrast, results based on CRU TS showed significant correlations with temperature. Based on these results, we anticipate that local mechanisms, such as altitude, orographic lift, aspect, and vegetation, are more responsible for precipitation than temperature. On the other hand, the ERA5-land climate with the highest spatial resolution among the chosen data sets showed a similar negative correlation for precipitation but an opposite (negative) correlation for temperature with δ^18^O_TRC_ chronologies (Fig. 4). The APHRODITE data set also showed the most robust relationship with the instrumental climate in both study sites, especially with precipitation (Fig. 7), as reported in a previous study by Li et al. (2018). Possible reasons for this may be that the interpolation of the APHRODITE data set is based on a denser network of climate stations than the CRU TS data set in the Himalayan region and that it is regionally calibrated for monsoon Asia (Yatagai et al. 2012). Previous studies assessing the performance of different data sets to capture observed climate patterns in the Himalayas showed spatial (regional) inconsistency. For example, the performance of the data set depended on elevation and the latitude (Chen et al. 2021; Kanda et al. 2020). The observed differences in data set performance likely arise from how these data sets are derived, including their spatial resolution, the density of observation networks, the interpolation and calibration methods used, and their ability to capture local climatic effects. Understanding these differences is crucial for selecting the appropriate data set for reliable climate impact studies.

### Relationship between climate and the pine isotope chronologies

Overall, the monthly (Fig. 4) and daily (Figs. 5 and 6) climate variables revealed a similar response of both δ^18^O_TR_ chronologies. In general, results for precipitation amount indicate higher importance in determining the isotopic signatures in the trees at both sites.

In the case of monthly climate, both of the pine isotope chronologies correlated negatively with precipitation of early summer monsoon season (May and June) and positively with summer temperature (particularly for June and July) (Fig. 4). In contrast, due to trees’ inconsistent early monsoon season response, late monsoon season precipitation (particularly for September) showed a stronger influence in the PIRO site than during the early monsoon. The 30-day averaged daily data also showed that the correlation between δ^18^O_TR_ and precipitation becomes significant from mid-May to the end of June and from mid-September to October. The negative correlation with early monsoon season precipitation is in line with findings from other conifers from Nepal (Brunello et al. 2019; Sano et al. 2011), India (Sano et al. 2017; Xu et al. 2018), and Bhutan (Sano et al. 2017). The significantly negative correlation with precipitation amount can be associated with the ‘amount effect’, which advocates that the greater rainfall intensity, the lower the δ^18^O value in the rainwater. In our sites, the summer monsoon season begins on average around 4 June (DOY 155) and generally persists until 18 or 19 September (DOY 261 or 262) (Fig. S4). Hence, an adequate amount of rainfall is apparent during these months. As shown in Fig. 2, the study sites remain comparatively drier before the summer monsoon onset, with low humidity and high temperature. The precipitation signal in May and June is more prominent because the drier atmospheric conditions result in enhanced ^18^O enrichment due to the evaporation of precipitation water before it reaches the ground. Further, since the pine trees have a shallow root system, they take up the soil water from the topsoil layer, which is generally enriched in ^18^O (Liu et al. 2010).

In contrast to the pre-monsoon season, the summer season in our study sites is characterised by maximum temperature and adequate precipitation (see Fig. 2). Trees open the stomata due to ample soil moisture availability, resulting in enhanced evaporative enrichment at the leaf level. As a result, the atmospheric moisture conditions (relative humidity and vapour pressure deficit) regulated by temperature determine the isotopic signature of the tree ring. This is also evident by a positive correlation with VPD on a monthly scale (Fig. 4). Interestingly, the correlations with the daily VPD and precipitation indicated a weaker relationship during both study sites’ peak monsoon season (July and August). The peak monsoon season is generally accompanied by a dense cloud cover, leading to high relative humidity and strongly reduced irradiation. We infer that such atmospheric conditions reduce the evaporative enrichment of the leaf water. On the other hand, the soil already becomes water-saturated during May and June, so any excessive rainfall during July and August immediately converts to surface runoff (Nepal et al. 2021) without being utilised by the trees.

### Temporal inconsistency between site chronologies and their climate relationships

Our findings indicated a strong overall correlation between the two δ^18^O_TR_ chronologies. However, a notable divergence occurred between the chronologies after 1985 when examining the running correlation. During this period, there was a significant decrease in inter-series correlation, indicating inconsistency even among individual trees. Conversely, an improved correlation was observed between the two Tree Ring Width (TRW) chronologies and an enhanced inter-series correlation after 1985, suggesting an increased coherence within site chronologies and among individual trees within each site. This convergence between TRW chronologies and the improved inter-series correlation supports the notion that the decoupling between the δ^18^O_TR_ chronologies is not a result of improper cross-dating but rather due to other external or internal factors. To test the consistency of the δ^18^O_TR_-climate relationships, we conducted 30 years of running correlations of δ^18^O_TR_ chronologies with monthly and daily climate data. We chose precipitation, temperature, relative humidity, and VPD from the CRU TS data set as the monthly climatic parameters, temperature and precipitation from the APHRODITE data set, and VPD from the ERA5-land data set for the daily climate.

In addition to decreasing coherency between PIWA and PIRO chronologies, climate-δ^18^O_TR_ correlations were also weakened after the 1990s, except for the early monsoon precipitation in PIWA and late monsoon precipitation in both sites. Similar decoupling in response was also reported by several other studies worldwide (An et al. 2019; Daux et al. 2011; Reynolds-Henne et al. 2007; Sarris et al. 2013; Seftigen et al. 2011; Szejner et al. 2018). Recent climate change was identified as the principal driving factor for such a decoupling (Savard and Daux 2020). Climate change indirectly results in such decoupling either by altering the timing of the monsoon season by hampering the air parcel movement to the sites or by modifying the local moisture conditions due to extended drought. On the other hand, there was no significant spatial and temporal variation of the monsoon season in our sites (Fig. S3 and S4). Throughout the studied interval, the onset and cessation of the summer monsoon were very similar (1-day delay) between the study areas, showing no significant temporal trends. This indicates no detectable monsoon seasonality difference between sites responsible for the observed isotopic decoupling between two isotope chronologies and their response to climate.

**Fig. 8:**
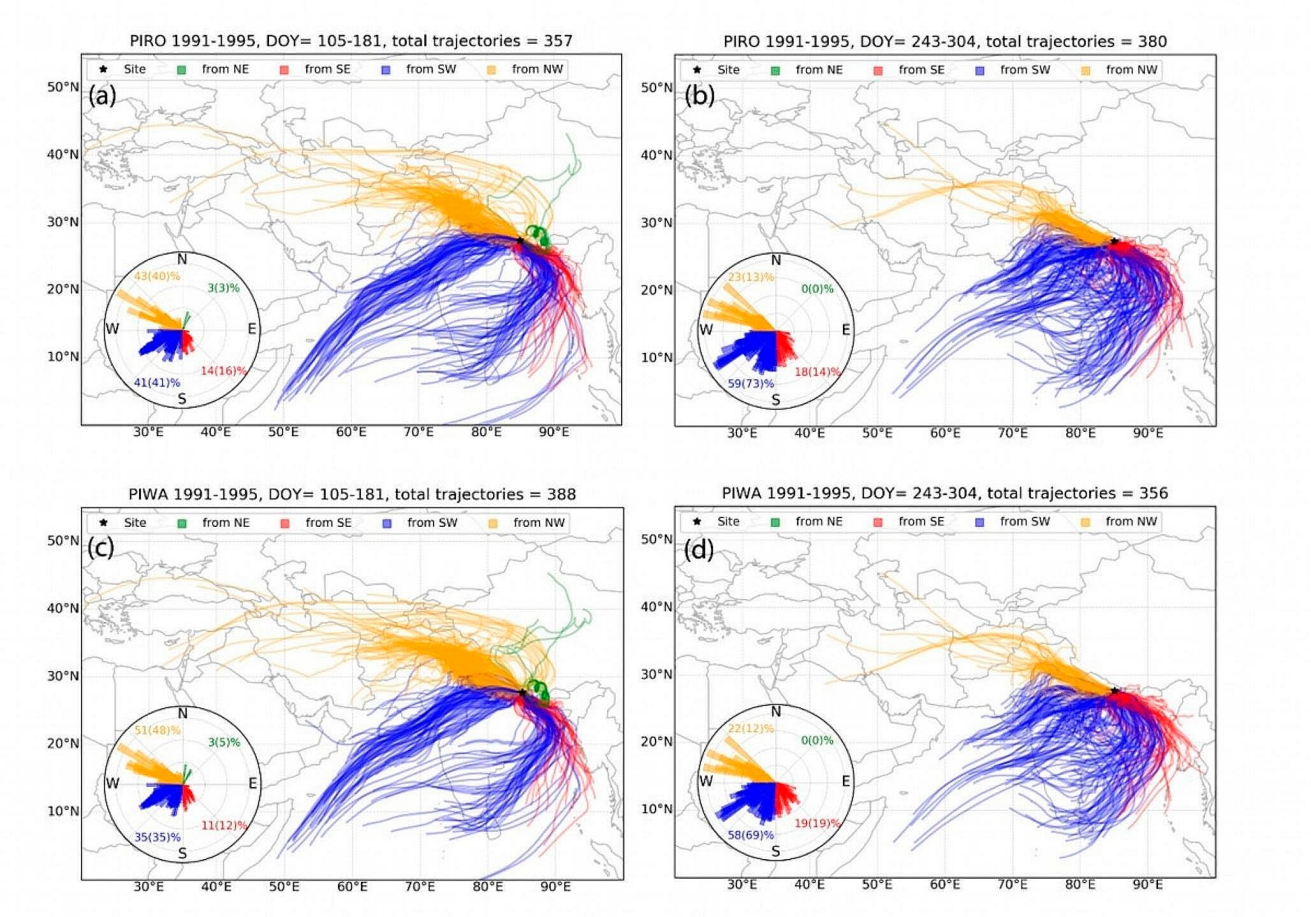
Air parcel trajectories to the sites from 1991–1995. The upper panels (**a** and **b**) show the trajectory path to the PIRO site for DOY 105–181 and DOY 243–304, whereas the lower panel (**c** and **d**) shows the same for the PIWA site. The polar plots inside each sub-figure indicate the percentage of trajectories from different directions (N: North, E: East, S: South, and W: West). The values in parenthesis represent the percentage of precipitation from each bearing (direction). (Please see Fig. S5-S8 in supporting documents for all analysed 5-year periods.)

**Fig. 9:**
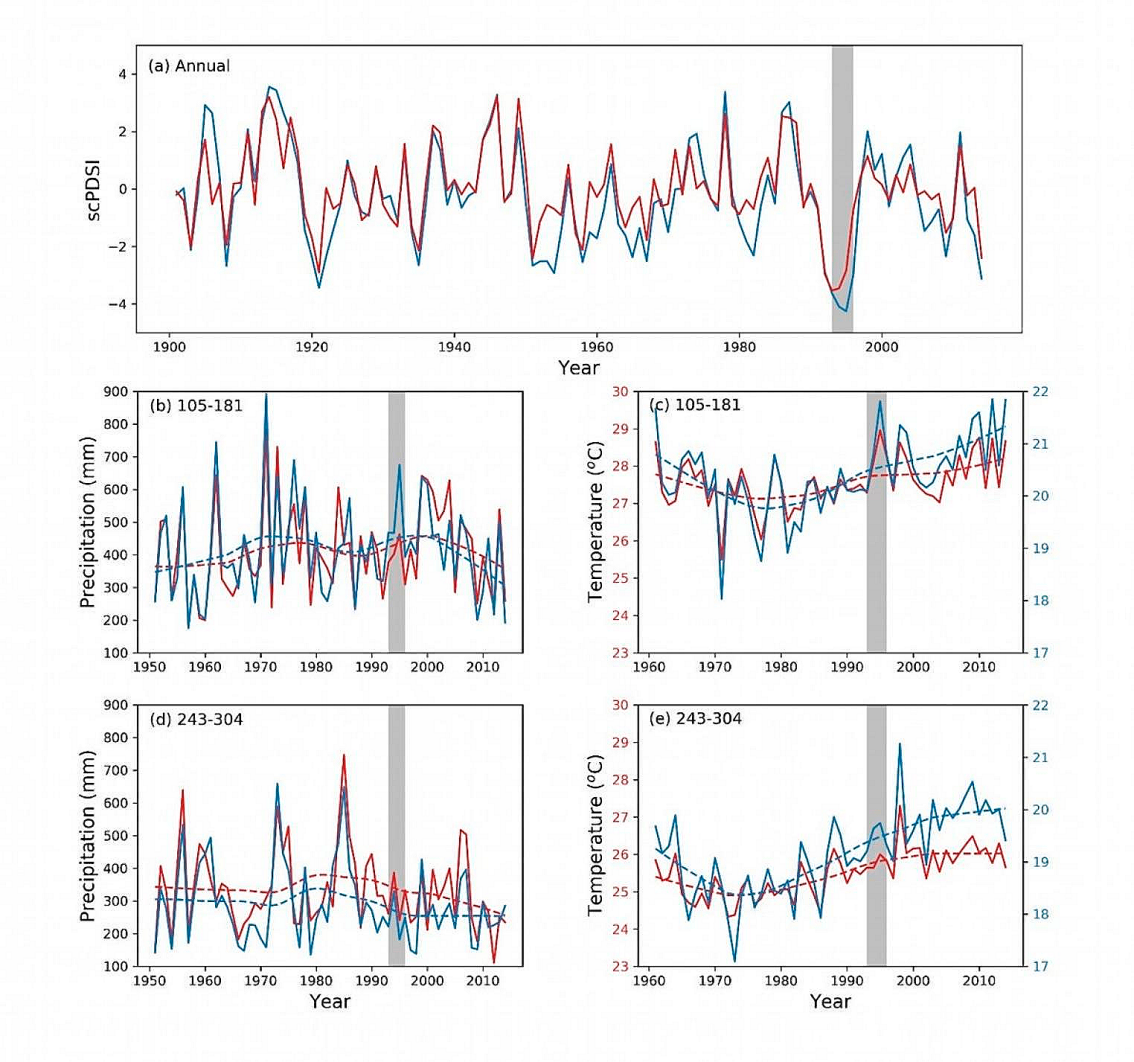
(**a**) Annual scPDSI of both investigation sides, indicating a severe drought during 1993–1995, indicated by grey bars in all panels. (**b**) and (**d**) total precipitation trends from days 105–181 (representing the early monsoon season) and 243–304 (= late monsoon season), respectively. (**c**) and (**e**) average temperature trends for the same periods. Red and blue colours represent the PIRO and PIWA sites. Dashed lines represent the locally weighted scatter plot smoothing (LOWESS) lines

The back trajectory analysis (BTA) showed similarities in air parcel flow direction to both study sites. The air parcels during the early monsoon season (DOY 105 to 181) were dominantly coming from the northwest (NW) direction, followed by trajectories from the southwest (SW) (Fig. 8 and S5-S8). On the other hand, most of the moisture during the late monsoon season (DOY 243 to 304) originated from SW. However, there was no significant change in the proportion of air parcels coming from a specific direction since 1981. This result advocates that changing moisture pathways or water vapour source areas does not cause isotopic decoupling. This makes us speculate that the divergent relation between the site chronologies in our study site is more likely due to the severe droughts during 1993–1995 CE in both locations (Fig. 9a).

Drought events were equally intense in all seasons (see Fig. S9 from the supporting document). In the southwestern USA, the divergent δ^18^O_TR_-VPD relationship was also related to increased drought events (Szejner et al. 2018). Our sites experienced a rapid surge in temperature and a departure of total precipitation after the drought event during the early monsoon period (Fig. 9b and c). The 7^°^C warmer climate in the PIRO site implies less soil moisture availability due to high potential evapotranspiration than the PIWA site. As a result, trees in the PIRO site take up water from deeper soil layers, which generally show less enriched isotope values (Liu et al. 2010). The enhanced drier conditions in the PIRO site after 1995 CE might have forced trees to reduce transpiration by closing stomata, resulting in negative relationships with temperature (Cernusak et al. 2019). It is noteworthy that the correlations between the two δ^18^O_TR_ chronologies became weaker after the drought event (Fig. 3c). This decoupling between the chronologies reflects that the isotopic signature of the whole tree ring cellulose is dominated by the cellulose formed during the early growing season, and is determined by the early monsoon season source water signal. A divergent δ^18^O_TR_-climate relationship due to the deepening of the soil water pool was also reported in *Larix decidua* from the French Alps (Daux et al. 2011) and from *Pinus halepensis* from the Mediterranean climate region (Sarris et al. 2013). Although the late monsoon seasons showed similar temperature and precipitation trends at our study sites (Fig. 9d and e), the atmospheric conditions (such as evapotranspiration) are drastically different. Since this season occurs immediately after the monsoon season, we anticipate wetter soils and a saturated atmosphere. The stronger late monsoon season correlation with climate in the PIRO site after the drought event in the 1990s also suggests that being an evergreen species, *P. roxburghii* might have shifted the moisture uptake partly into the late monsoon season. This further implies that in the PIWA site, the tree ring cellulose imprints mixed signals of moisture during early monsoon (mostly from NW) and late monsoon (mainly from SW), but the signal in the PIRO site is dominated by the isotope signal of late monsoon precipitation (mostly from SW).

## Conclusions

We developed two statistically robust δ^18^O_TR_ chronologies for two native pine species (*Pinus roxburghii* and *Pinus wallichiana*) in the Nepalese Himalaya. The δ^18^O_TR_ chronologies showed significant differences caused by elevation differences. Both chronologies were highly correlated until the beginning of the 1990s. Afterwards, decreasing correlations occurred. We found variations in climate response of both species for climate data sets from different sources at various spatial and temporal resolutions. The APHRODITE data set, specifically calibrated for and from the South Asian Summer Monsoon (SASM)-dominated region, outperformed others, especially in the case of precipitation. Like previous studies on high mountain conifers, the isotope chronologies of the two congeneric species showed a similar climate response: negative correlations with monsoon precipitation and positive correlations with temperature. However, daily resolved climate input data disentangled the statistically insignificant relation during the peak monsoon season. In the peak monsoon season (July-August), δ^18^O_TR_ was controlled by the meteoric moisture (relative humidity and vapour pressure deficit), indicating enhanced leaf water discrimination. We observed no significant change in the air parcel trajectories from a specific direction between the two study sites over the entire study period, indicating no change in the moisture source for both sides. In addition, the monsoon season length also showed similarities between the sites in terms of both duration (onset and cessation) and long-term trends. As we anticipated, the strength of the influence of regional climate was significantly controlled by the local climate conditions, with a stronger influence in the high-elevation site to nullify the regional drought effect.

Even though *P. roxburghii* is considered a stress-tolerant species, the drought during 1993–1995 posed an adverse effect on its physiology, resulting in isotopic decoupling in contrast to the PIWA site. This infers that the local temperature and moisture availability strongly regulate the impact of regional extreme climate events such as drought on tree-ring oxygen isotope variations. Our results advocate that the isotopic decoupling can hamper long-term climate reconstructions by switching or weakening the proxy-target response. Implementing climate data with optimum spatial and temporal resolution can enhance the δ^18^O_TR_-climate relationships on the intra-annual scale. On the other hand, climate data with lower spatial resolution can buffer the site-specific local environmental effects. Hence, evaluating different climate data sets with available station data is essential before using them in tree-ring climate response analyses. In addition, understanding the tree responses to climatic changes can guide forestry and conservation practices. For instance, it can help to select species and varieties more resilient to climate change, particularly in regions susceptible to droughts or extreme weather events.

### Electronic supplementary material

Below is the link to the electronic supplementary material.

Supplementary Material 1
