# Supplementary material for: Drought, temperature, and moisture availability: understanding the drivers of isotopic decoupling in native pine species of the Nepalese Himalaya
Source: Int J Biometeorol. 2024 Mar 5;68(6):1093–108. doi: 10.1007/s00484-024-02647-z (PMC11108894; doi:10.1007/s00484-024-02647-z)
Supplement: Supplementary file 1 — Supplementary Material 1 [file 484_2024_2647_MOESM1_ESM.docx]

Drought, Temperature, and Moisture Availability: Understanding the Drivers of Isotopic Decoupling in Native Pine Species of the Nepalese Himalaya

Sugam Aryal^1^, Jussi Grießinger^1^, Narayan Gaire^2^, Tribikram Bhattrai^3^, Achim Bräuning^1^

^1^Friedrich‐Alexander‐Universität Erlangen‐Nürnberg, Institute of Geography, Wetterkreuz 15, 91058 Erlangen, Germany

^2^Department of Environmental Science, Patan Multiple Campus, Lalitpur, Nepal

^3^Central Department of Biotechnology, Tribhuvan University, Kathmandu, Nepal


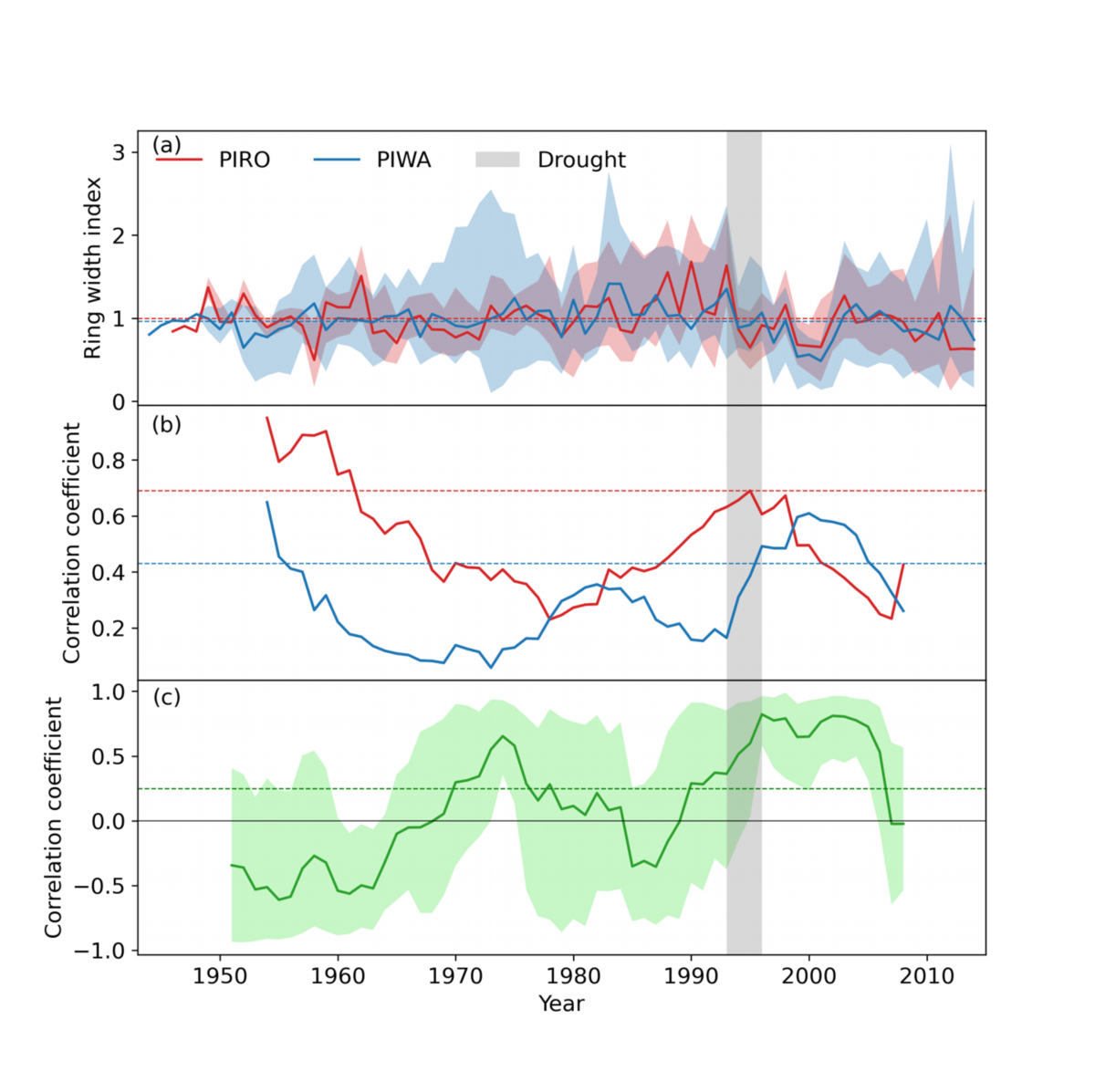


Fig. S 1 (a) The tree ring width chronologies of P. roxburghii and P. wallichiana, including the 5% confidence intervals and mean values represented by dashed colored lines. (b) The 11-year moving means inter-series correlation of the tree ring width series. (c) The 11-year running bootstrapped correlation between the PIRO and PIWA tree ring width chronologies with a 5% confidence interval.


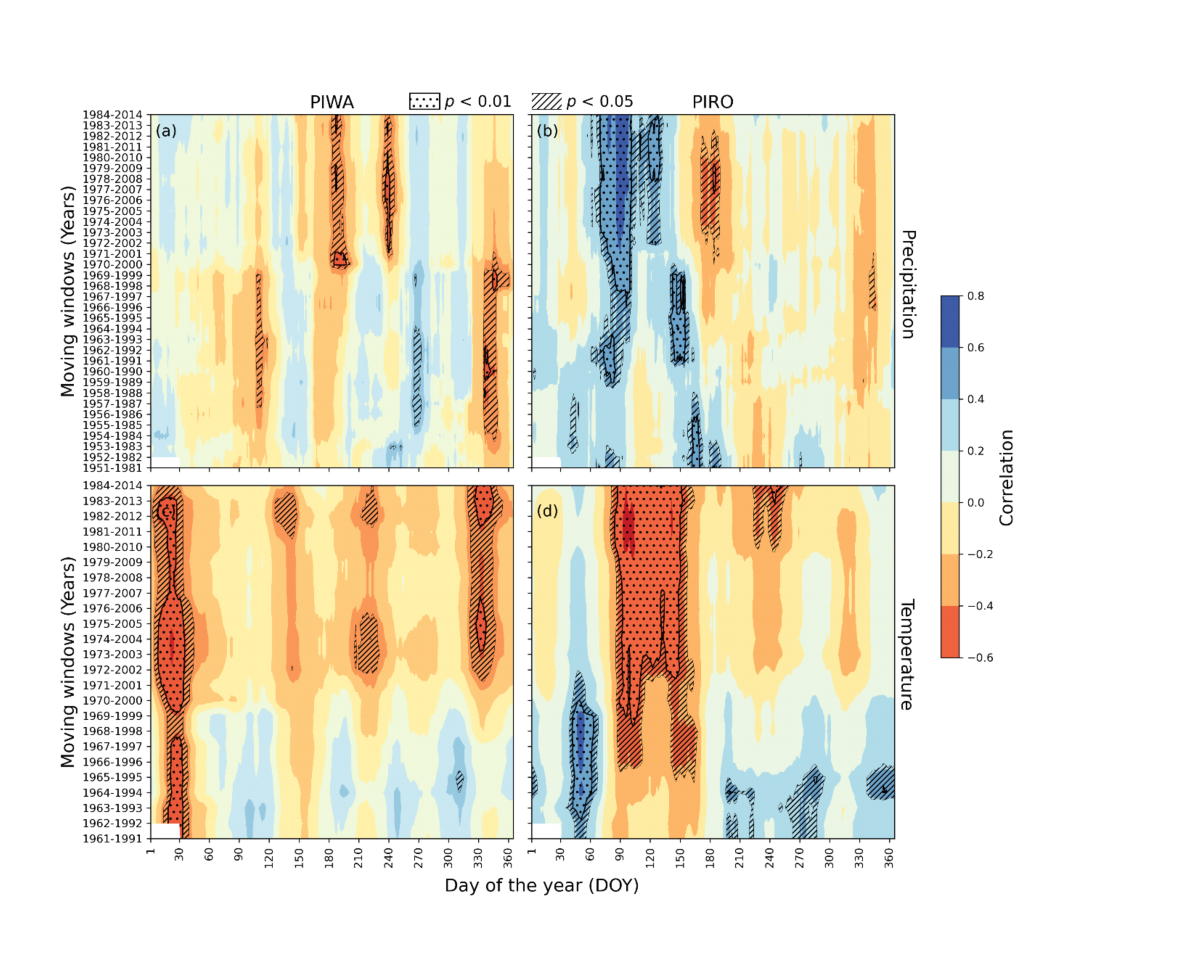


Fig. S 2 30-year moving correlation analysis of PIWA and PIRO tree ring width chronologies using a 30 days moving climate windows. Figures (a) and (b) represent correlations with running sums of daily precipitation, (c) and (d) represent correlations with a running mean of daily temperatures. Shaded areas represent significant correlations at p<0.01(dotted area) and p<0.05 (hatched area).


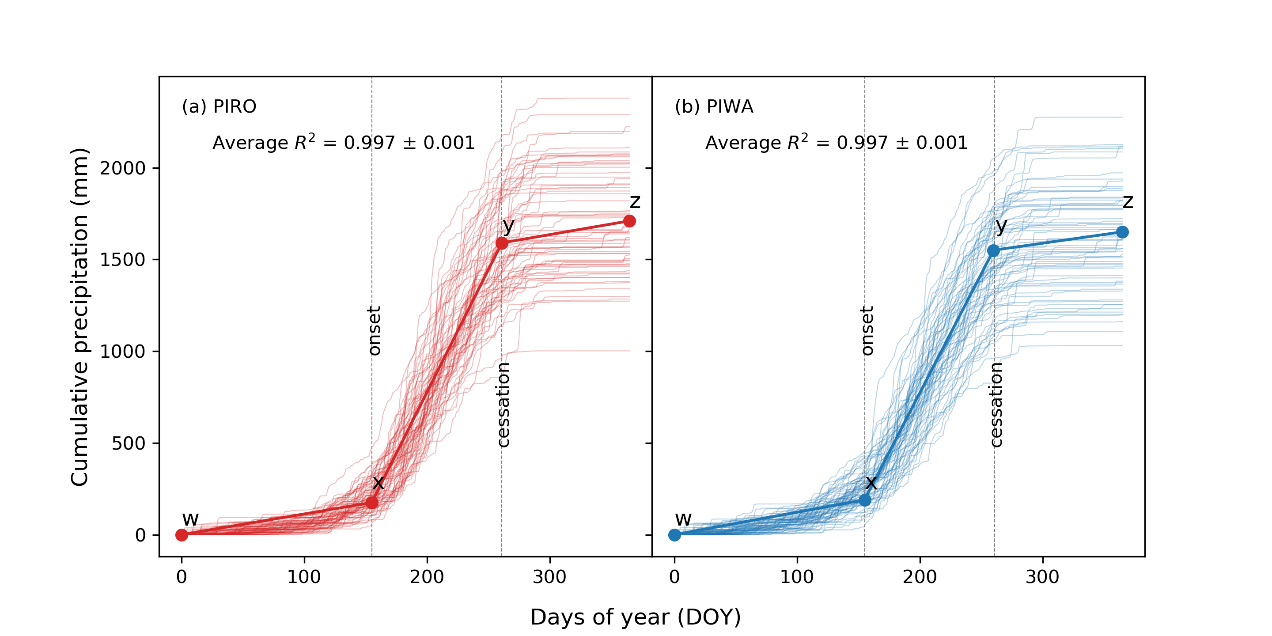


Fig. S 3 Cumulative precipitation for (a) PIRO and (b) PIWA sites extracted from the APHRODITE dataset. The exemplary line segments wx, xy, and yz were used for piecewise regression. R^2^ represents the average regression coefficient of piecewise regression.


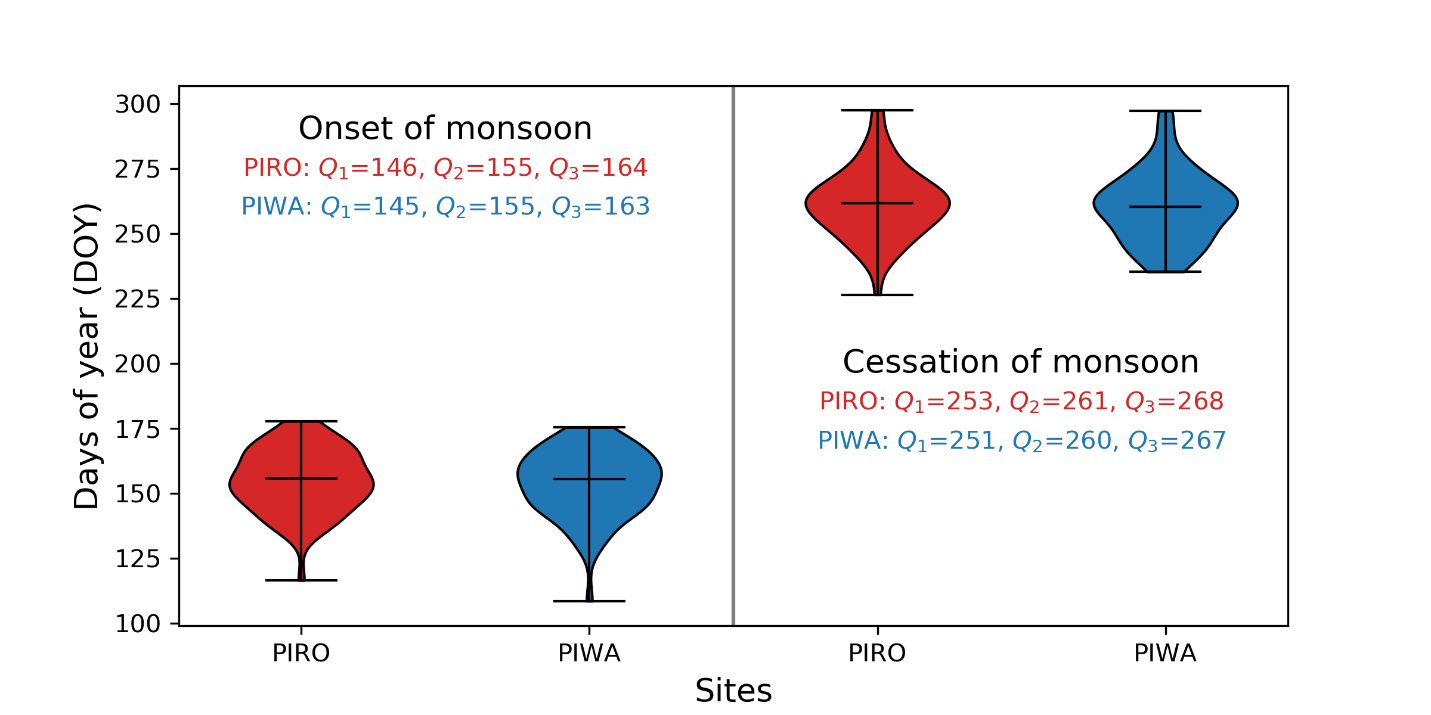


Fig. S 4 The onset and cessation of the summer monsoon season in PIRO and PIWA sites. Q_1_, Q_2_ and Q_3_ represent the first, second and third quartile.


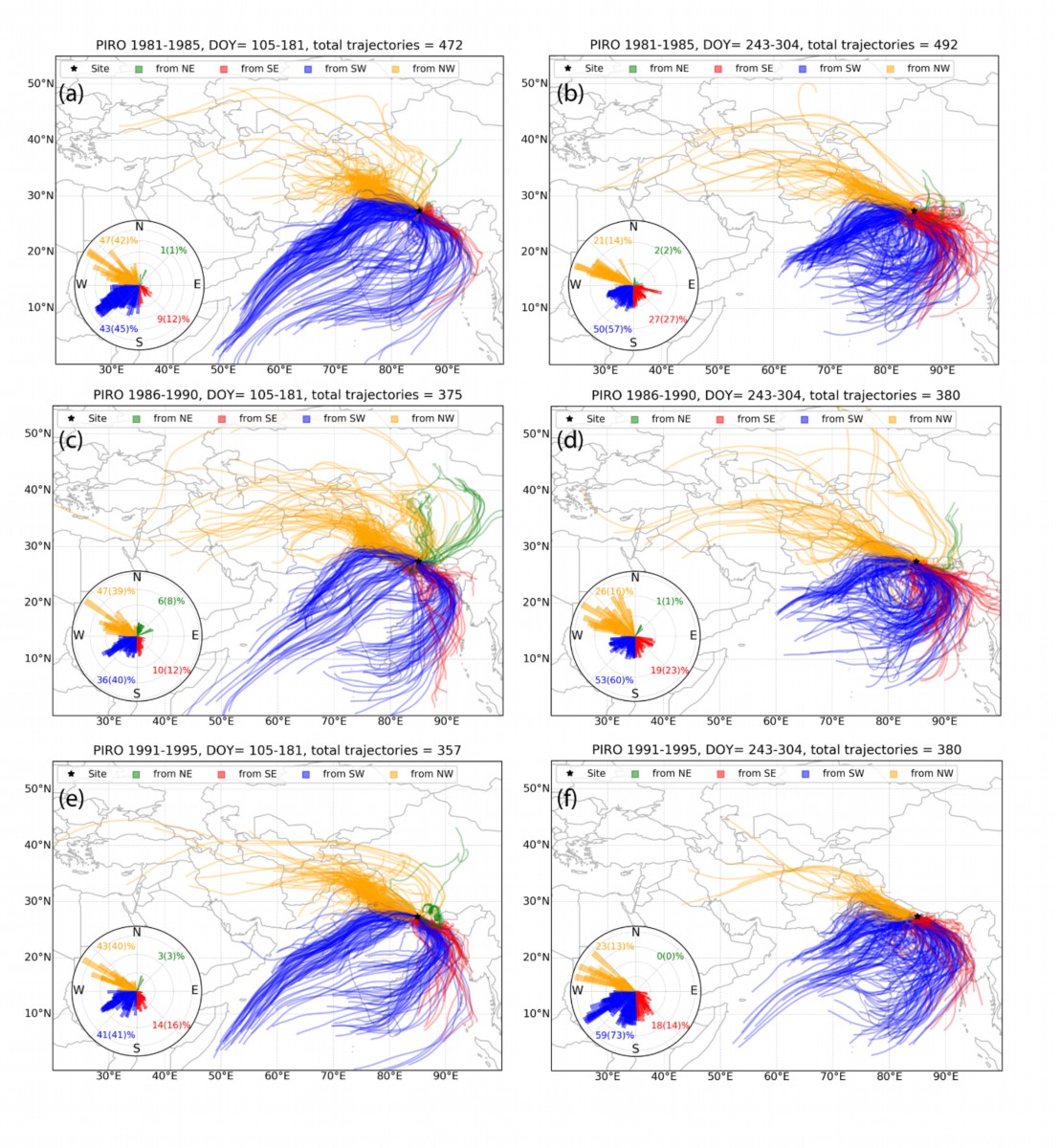


Fig. S 5 Air parcel trajectories to the PIRO site from 1981-1995. The left panels (a,c, and e) show the trajectory pathways for DOY 105-181, whereas the right panels (b,d, and f) show the same for DOY 243-304. The polar plots inside each sub-figure indicate the percentages of trajectories from different directions (N: North, E: East, S: South, and W: West). The values in parenthesis represent the percentage of precipitation from each bearing (direction).


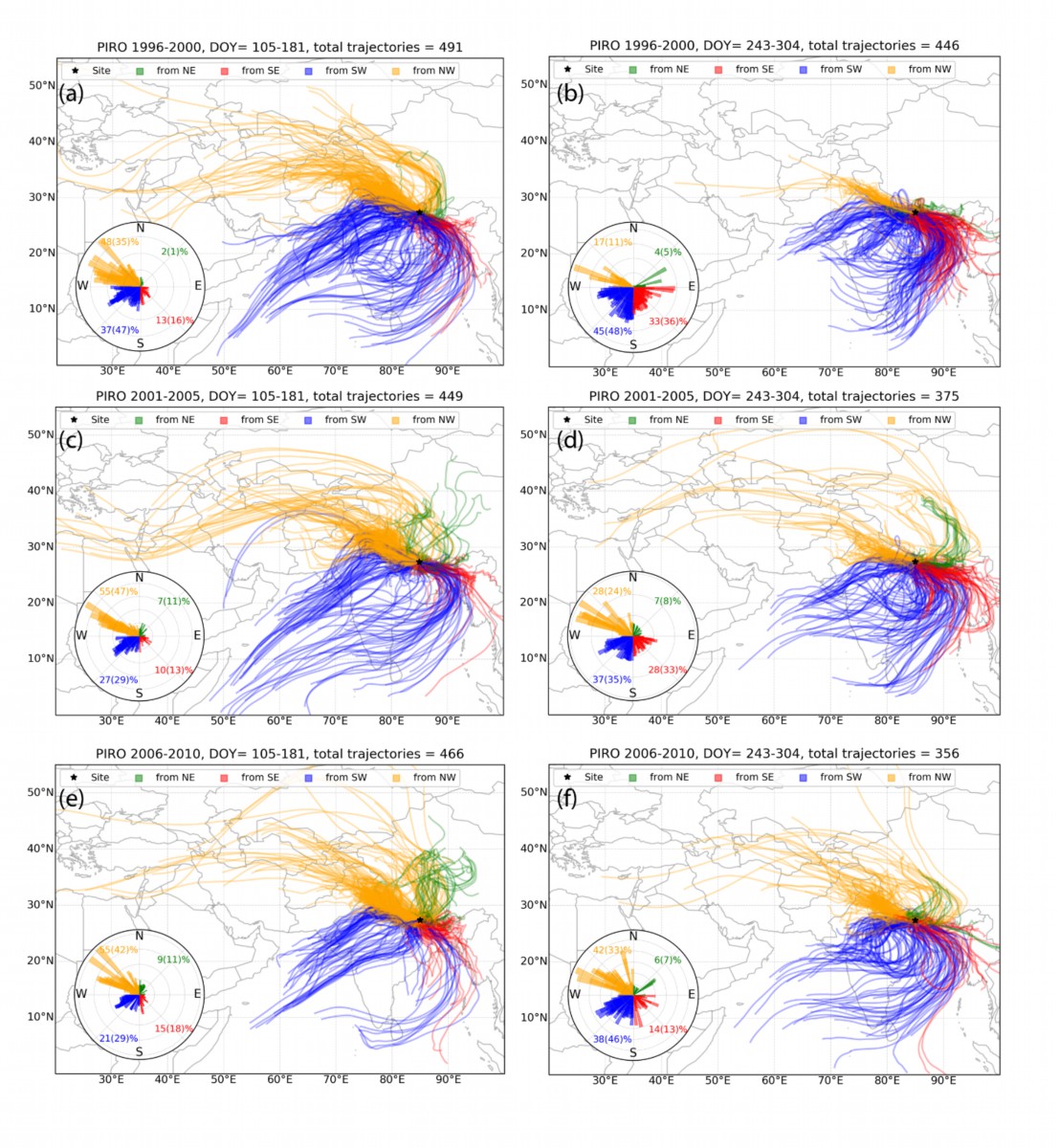


Fig. S 6 The air parcel movement to the PIRO site for the period 1996-2010. The left panel (a,c and e) shows the trajectory path for DOY 105-181, whereas the right panel (b,d and f) shows the same for DOY 243-304. The polar plot inside each sub-figures indicates the percentage of trajectories from different directions (N: North, E: East, S: South, and W: West). The value in parenthesis represents the percentage of precipitation from each bearing (direction).


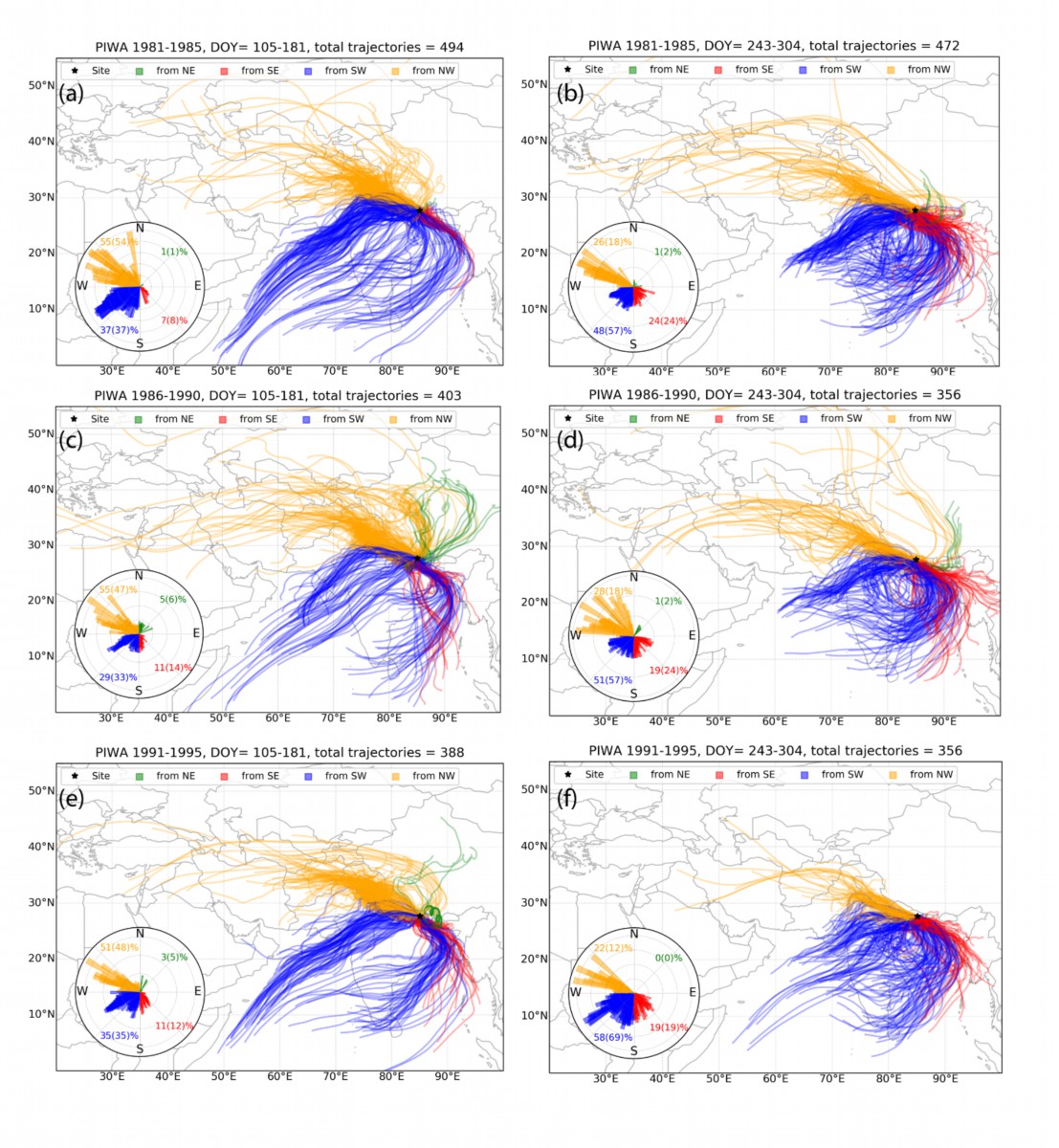


Fig. S 7 The air parcel movement to the PIWA site for the period 1981-1995. The left panel (a,c and e) shows the trajectory path for DOY 105-181, whereas the right panel (b,d and f) shows the same for DOY 243-304. The polar plot inside each sub-figures indicates the percentage of trajectories from different directions (N: North, E: East, S: South, and W: West). The value in parenthesis represents the percentage of precipitation from each bearing (direction).


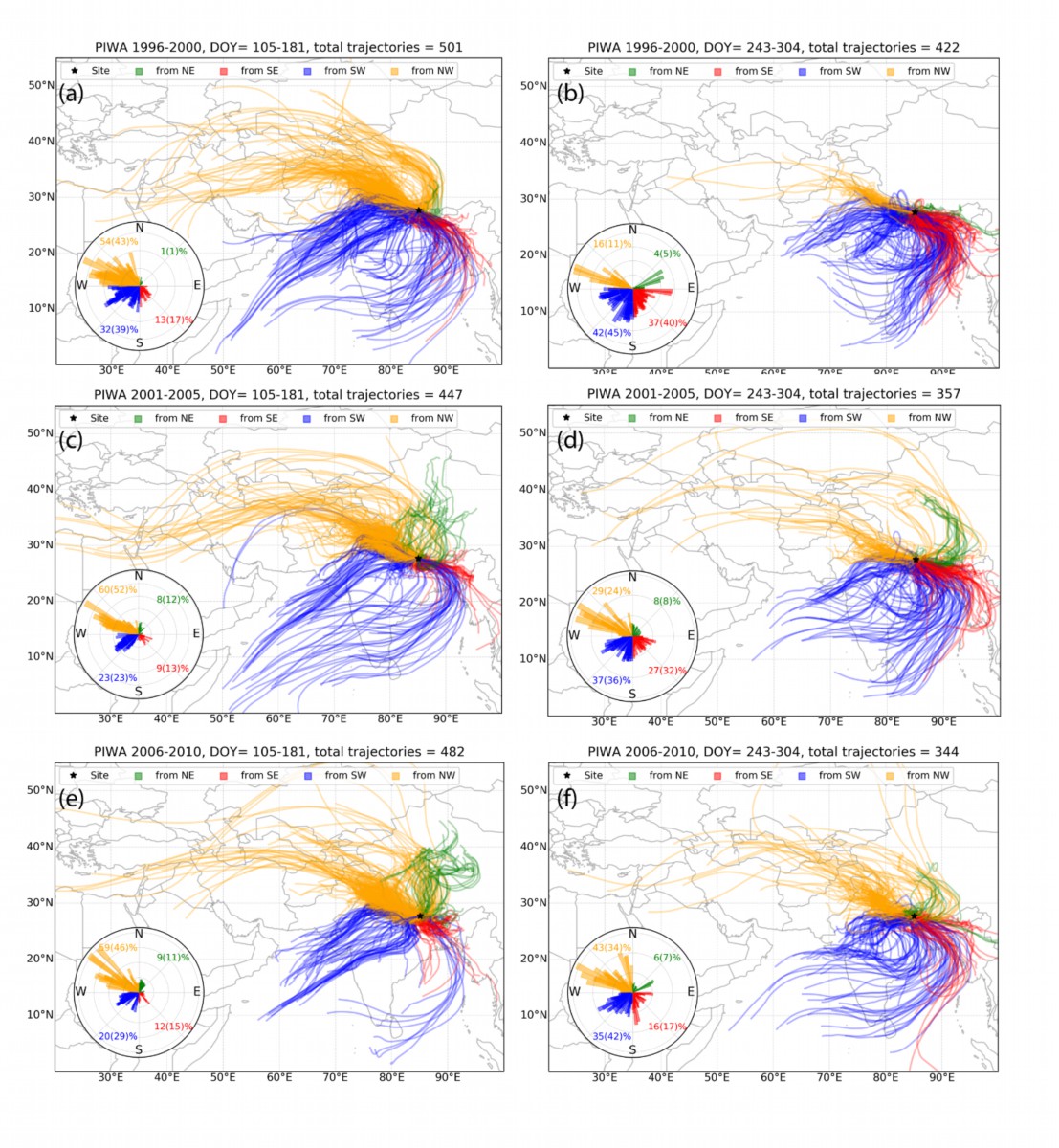


Fig. S 8 The air parcel movement to the PIWA site for the period 1996-2010. The left panel (a,c and e) shows the trajectory path for DOY 105-181, whereas the right panel (b,d and f) shows the same for DOY 243-304. The polar plot inside each sub-figures indicates the percentage of trajectories from different directions (N: North, E: East, S: South, and W: West). The value in parenthesis represents the percentage of precipitation from each bearing (direction).


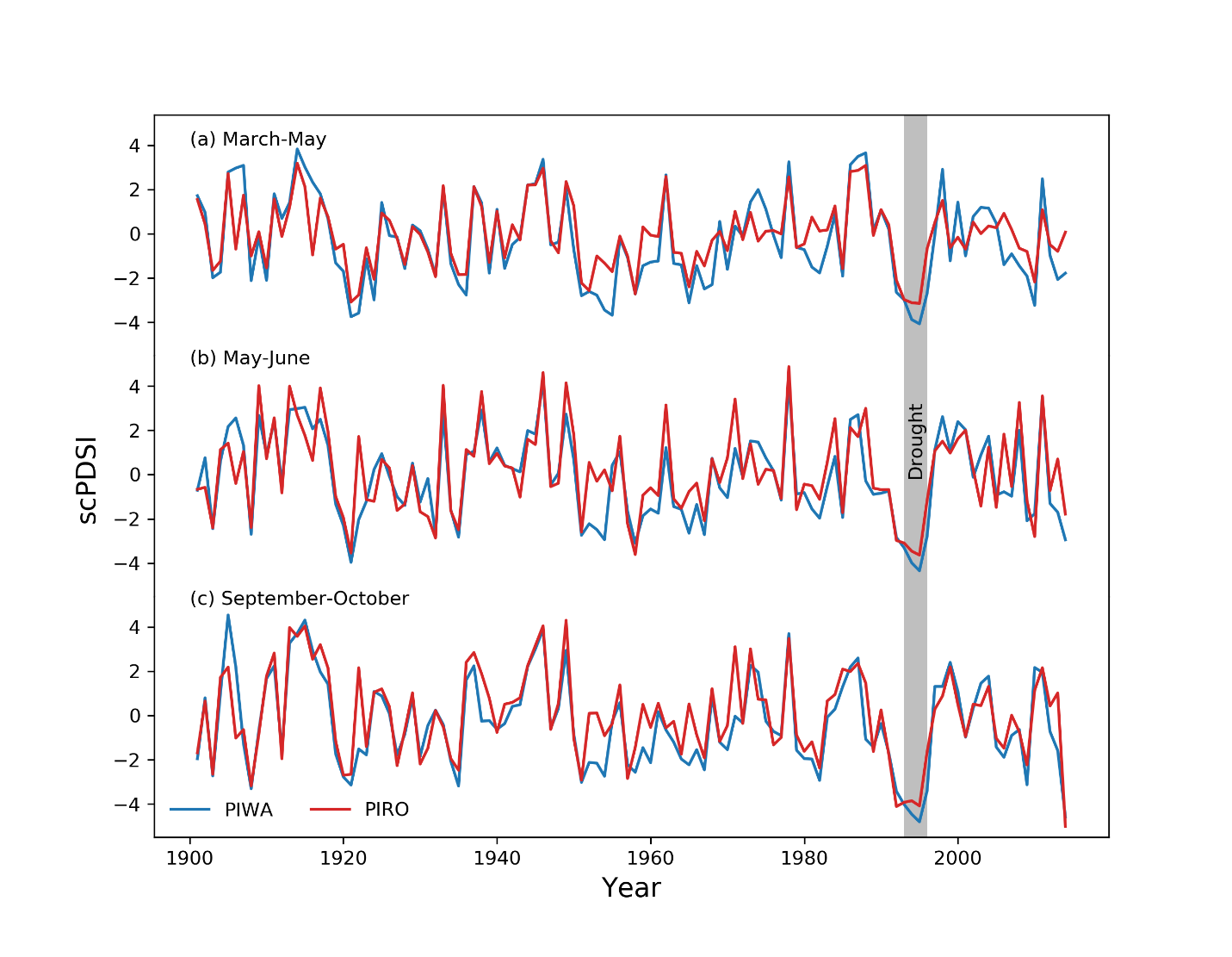


Fig. S 9 Seasonal scPDSI series at both study sites (PIWA, PIRO) extracted from CRU data. The grey bar indicates the 1990's drought period (1993-1995).
